# Nano-molecularly imprinted polymers (nanoMIPs) as a novel approach to targeted drug delivery in nanomedicine

**DOI:** 10.1039/d1ra08385f

**Published:** 2022-02-01

**Authors:** Konstantin G. Shevchenko, Irina S. Garkushina, Francesco Canfarotta, Sergey A. Piletsky, Nickolai A. Barlev

**Affiliations:** Institute of Cytology RAS St. Petersburg Russia nick.a.barlev@gmail.com; Institute of Biomedical Chemistry RAS Moscow Russia; Institute of Macromolecular Compounds RAS St. Petersburg Russia; MIP Diagnostics Colworth Park, Bedfordshire UK; University of Leicester Leicester UK

## Abstract

Molecularly imprinted polymers – MIPs – denote synthetic polymeric structures that selectively recognize the molecule of interest against which MIPs are templated. A number of works have demonstrated that MIPs can exceed the affinity and selectivity of natural antibodies, yet operating by the same principle of “lock and key”. In contrast to antibodies, which have certain limitations related to the minimal size of the antigen, nanoMIPs can be fabricated against almost any target molecule irrespective of its size and low immunogenicity. Furthermore, the cost of MIP production is much lower compared to the cost of antibody production. Excitingly, MIPs can be used as nanocontainers for specific delivery of therapeutics both *in vitro* and *in vivo*. The adoption of the solid phase synthesis rendered MIPs precise reproducible characteristics and, as a consequence, improved the controlled release of therapeutic payloads. These major breakthroughs paved the way for applicability of MIPs in medicine as a novel class of therapeutics. In this review, we highlight recent advances in the fabrication of MIPs, mechanisms of controlled release from the MIPs, and their applicability in biomedical research.

## Abstract 2

The review highlights the recent advances in fabrication of molecularly imprinted polymers and their applicability for drug delivery.

## Graphical abstract

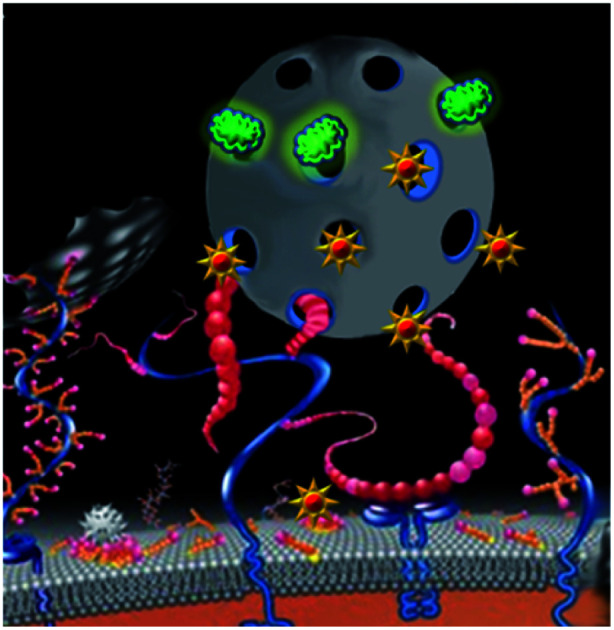

## Introduction

Antibodies play an active role in the immune system through recognition and inactivation of pathogens. With a growing understanding of the benefits brought by precision therapy, monoclonal antibodies were successfully exploited for targeted drug delivery. The examples range from antibody–drug conjugates to functionalized nanoparticles. Despite obvious advantages the antibodies are expensive in development and production which significantly hinders their availability for the patients. Another limitation of monoclonal antibodies is their general inability to recognize small, denatured, or slightly altered peptide antigens. These shortcomings stimulate the search for the chemical mimics of antibodies that would function by the same “lock and key” principle, but would be inexpensive in production. The recent breakthrough in the development of molecularly imprinted polymers (MIPs) against peptide and protein targets has made them one of the key candidates to replace antibodies.^1^

**Fig. 1:**
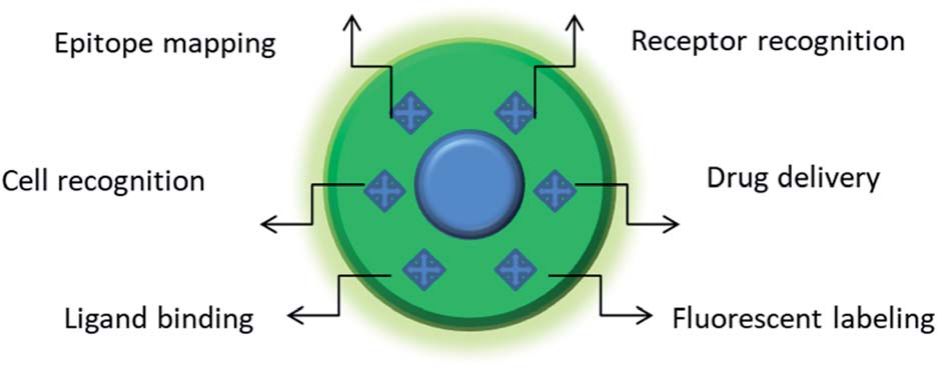
Featured areas of nanoMIP applications in biomedicine. Depending on the composition and nature of imprinted structure(s), MIPs may serve as a powerful platform for biosensing, molecular therapy, and the development of new tools for cellular studies. For instance, MIPs imprinted against particular cell surface markers can distinguish between different types of cells with different expressions of this marker. Furthermore, the use of MIPs against a particular protein allows its quick epitope discovery, whereby MIP-bound regions of the protein are protected against trypsin digest and the unprotected regions are subject to degradation.^5^ The MIP-protected peptide sequences are subsequently identified by mass-spectrometry. MIPs fabricated against cell surface receptors can be used for the targeted delivery of drugs. MIPs bound to cell surface markers can label whole cells. MIPs can also prevent the ligand binding to their receptors, thereby affecting the physiology of cells.

Nano-MIPs may be functionalized with small molecules and fluorescent dyes, which makes them a powerful tool for drug delivery (Fig. 1). They are characterized by high drug loading capacity, specificity of a payload delivery and versatility of molecular targets.^2^ However, their most important property for potential clinical application is their excellent biocompatibility.^3^ The use of MIPs may help to bypass the major flaws of nanocarriers – the fast blood clearance and potential toxicity.^4^

## Molecular basis for MIP mediated drug delivery

The recent developments of new approaches to the solid-phase synthesis of MIPs have minimized the batch-to-batch variation of nanoMIPs properties. This advancement resulted in a rapid growth of their biomedical applications. Initially, the research in this area was focused on the development of new synthesis methods and the employment of novel polymer classes. The latter aimed to develop new coatings that would be more effective in recognition of the target molecule and more precise in the grade of payload release. One of the most noticeable shifts prior to the solid phase synthesis was the transition from the use of covalent bindings to non-covalent bindings between the polymer and the template. Hydrogels based on derivatives of acrylic acid-based monomers (MAA, HEMA, DMAA) became a popular system for imprinting of small molecules, peptides and proteins as they retain the binding specificity even in aqueous solutions (for example, MIPs for sulfosalazine^6^ and *S*-amlodipine^7^). Cyclodextrin derivatives were another important material for MIPs fabrication, which have been used for the delivery of vancomycin and l-DOPA.^8–10^ However, a low cross-linker density reduced the stability of MIPs and required a more complex polymer network design. Concurrently, a high degree of water content significantly increased the biocompatibility of such nanoparticles, by decreasing protein absorption on their surface. Hence it reduced the likelihood of immune response, which is an important issue in the field of targeted drug delivery.^11,12^

The first report on the potential use of MIPs for the drug delivery was published in 1998. The polymer was built using methacrylic acid (MAA) cross-linked by ethylene glycol dimethacrylate (EGDMA). It was able to recognize and selectively distinguish between the anti-asthmatic drug theophylline and structurally similar caffeine. Importantly, these MIPs were capable of gradual release of theophylline. This study indicated that the selective binding characteristics of imprinted polymers could be used for development of the novel drug dosage forms.^13^ It has also set the stage for an *in vivo* application of MIPs. While MIPs have already been employed as a tool for biosensing and molecular recognition,^14–16^ their repurposing was a way to a shift in nanomedicine.

The first application of MIPs for drug targeting came in 2000 as an anecdotal result of experiments on protection of imprinted acrylic MIPs from excessive hydration in water solutions. This study has led to the development of different strategies for the spatial limiting of drug release, such as multilayer MIPs. They were capable of transdermal delivery of propranolol. The two layers consisted of cross-linked EGDMA–MAA which carried the imprinted compound and a non-polar transdermal adhesive.^17,18^ Another approach exploited soft contact lenses from polymeric hydrogels for MIP-mediated delivery. In the pioneering paper, testosterone was added to the poly(2-hydroxyethyl methacrylate) (pHEMA) mixture during synthesis. Upon polymerization, testosterone gradually released from the polymers into the aqueous solution.^19^ Next, the soft contact lenses have been extensively used for the delivery of enorfloxacin,^20^ prednisolone,^21^ timolol,^22,23^ ciprofloxacin, and ketotifen.^24^

For life science applications, MIPs should specifically and efficiently recognize proteinaceous targets. To this end, polyacrylamide nanoparticles bearing imprints of short hydrophilic peptides were developed. The 28 nm size nanoparticles were synthesized against a peptide derived from the green fluorescence protein (GFP) using the microemulsion method whereby the aqueous solution of monomers and the peptide target are added to the mixture of nonpolar solvents.^25^ In another study, 37 nm nanoMIPs were synthesized against the short peptide sequence CNCKAPETADCAFVCFLS, which partially corresponded to the immunogenic epitope of the mitochondrial/cell surface p32 protein, which is often overexpressed in cancer cells. The MIPs remained stable and functionally active even in blood serum. They effectively bound the over-expressed target protein on the surface of 4T1 and BxpC-3 breast cancer cells *in vitro*. The developed nanoparticles also effectively targeted the BxpC-3 cells *in vivo* in mouse xenografts and specifically accumulated in the p32-positive cells. Finally, when loaded with methylene blue, these MIPs were successfully used for the photodynamic-based therapy of the tumour. This seminal work was one of the first to demonstrate the effective use of molecular imprinted polymers for targeted tumour therapy.^26^ Another frequently overexpressed tumour antigen is EGFR. The EGFR N-terminal nonameric peptide modified with palmitic acid was used as the imprinting template. The core–shell carbon dots functionalized with the developed MIPs specifically recognized the EGFR protein on the surface of HeLa cells both in cell culture and in mice as a xenograft model. Although the primary aim of the study was the development of new instruments for tumour sensing and imaging, the obtained data also suggested the usability of such nanoMIPs in drug delivery.^27^ Similar particle design has been used for targeting the C125 antigen by the doxorubicin-loaded MIPs. However, in this case the effective drug delivery has been demonstrated only *in vitro*.^28^ Analogous core–shell nanoparticles with a graphene oxide quantum dot core and a MIP shell were successfully employed for the light-controlled delivery of doxorubicin in bacteria.^29–31^ Collectively, the documented success of this approach suggests that such peptide-recognizing nano-MIPs with the size below 100 nm may not only substitute cellular receptors but can also be used as specific nano-carriers for the delivery of drugs.

## Biocompatibility

One of the critical parameters of precision nanomedicine relies on the biocompatibility of drug delivery systems, *i.e.*, they should have no adverse effects on healthy cells and tissues. The biocompatibility of MIPs primarily depends on the surface chemistry of the nanoparticles and requires their initial evaluation *in vitro*. New materials are usually tested for biocompatibility *in vitro* using the standard fibroblast-like cell line NIH/3T3.^32–36^ Generally, the analysis of toxic effects is based on whether or not nanoparticles lead to cell death. However, the concept of cytotoxicity should be linked to several other aspects, such as inflammatory response, alterations in the natural morphology or functions of the cell, overall effects on the cellular metabolism. Although not much information on the toxicity of MIPs is available, there are several examples indicating that nanoMIPs are usually not toxic. For instance, nano-MIPs fabricated from methacrylic acid and ethylene glycol dimethacrylate for the targeted delivery of olanzapine to the central nervous system showed low cytotoxicity with respect to the NIH/3T3 cell line.^33^ Furthermore, nano-MIPs based on trimethylolpropane trimethacrylate (TRIM), EGDMA and MAA co-polymers synthesized against melamine did not display cytotoxicity in HaCaT and HT1080 cells (keratinocyte and fibrosarcoma cell lines, respectively).^37^ The nano-MIPs based on poly(lactide-*co*-glycolide) (PLGA) and acrylic acid demonstrated complete biodegradation in cell medium.^38^ Thus, with the correct monomer composition, it is possible to generate biocompatible nano-MIPs that do not elicit cytotoxicity at least *in vitro* when tested against various cell lines.

Obviously, once MIPs would become more widely used in *in vivo* applications, more rigorous studies on their long-term *in vivo* toxicity would be necessary. It is worth mentioning that nanoparticles might not lead to apparent acute *in vivo* toxicity in the first instance, however they could potentially accumulate within organs. Thus, extensive studies are needed to understand the long-term effects of nanosystems due to their accumulation in various organs (liver, lungs, spleen, kidneys *etc*). Once nanoparticles enter the organism, they undergo several biological transformations. Typically, nanoparticles in the bloodstream get coated by opsonins and then sequestered in the reticuloendothelial system (RES), in order to be destroyed.^39^ However, if they are not biodegraded, nanoparticles may accumulate within cells and tissues with potential toxic effects. Therefore, long-term biocompatibility studies are required, in order to clarify the potential risk arising from particle accumulation. One approach to reduce their cellular internalization and to increase biocompatibility relies on the modification of the particle surface with neutral hydrophilic polymers. PEG with MW N 2000 Da is a particularly useful modifying agent for increasing the time of blood circulation of nanoparticles, due to its ability to reduce the adsorption of opsonins by means of steric repulsion forces.^40^

In conclusion, there is a potential for use of nano-MIPs *in vivo*, for drug delivery, imaging and diagnostic applications. However, long term evaluation of the toxicology of these materials is required before their practical application commences.

## Mechanisms of controlled drug release from MIPs

**Fig. 2:**
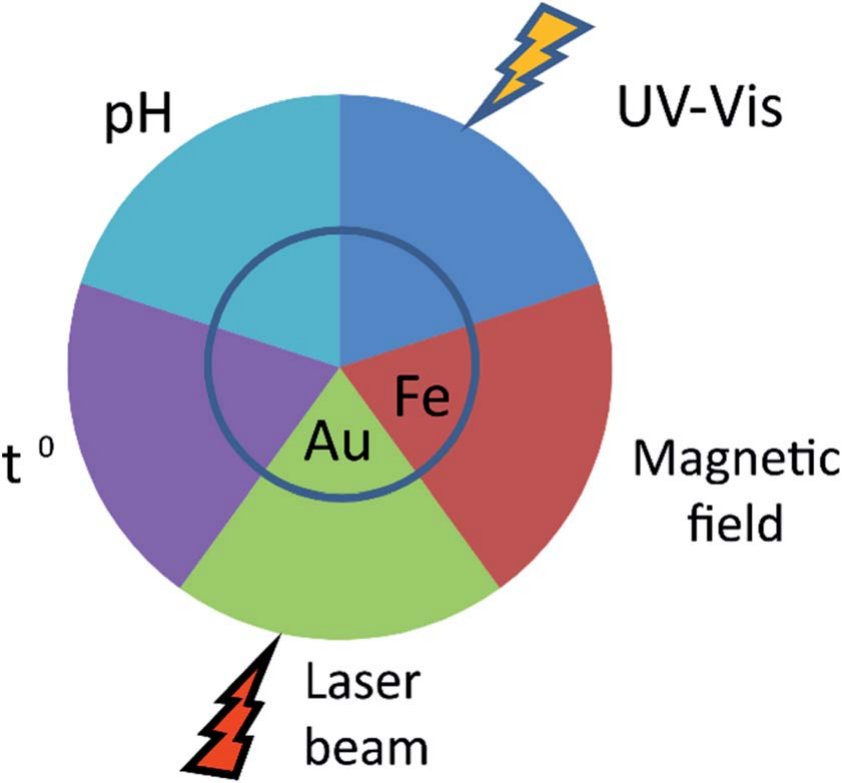
Mechanisms of controlled drug release from MIPs. Shown are various physical means for the controlled drug release from MIPs *in vivo*. The drug release from the carrier may be mediated by temperature (*T*) and acidity (pH). Introduction of plasmonic or magnetic particles (gold (Au) and iron oxide (Fe_2_O_3_ or Fe_3_O_4_), respectively) into the polymer core allows for the unload to be controlled by external magnetic or light fields (laser beam). Encapsulation of azobenzenes allows their release with UV-irradiation (UV-Vis).

The applicability of nanoparticles as nanodevices that are able to controllably release drugs *in vivo* relies on the nature of the release stimulus (Fig. 2). This is usually defined by the method of nanoparticle administration. An important feature of MIPs is their versatility with respect to routes of administration. Initially, studies on the controlled release of drugs from nanoparticles were focused upon their oral administration. The latter is undoubtfully, a more convenient and tolerable way of delivery for patients and could be applied to a variety of dosage forms. NanoMIPs however can do much more than just protect drug cargo from destruction in digestive track.

### pH sensitivity

The release of an imprinted molecule in response to changes in the acidity of the surrounding medium is one of the key features required for the effective design of therapeutic agents for oral delivery. For example, pH-dependent drug release may be suitable for the treatment of certain cardiovascular pathologies that are associated with changes in the acidity of blood serum. Such an effect can be based on changes in the physical volume of hydrogel due to solvation of its side chains upon changes in acidity. Such a possibility has been explored by using 20–63 micron size MIPs for peroral administration fabricated from EGDMA cross-linked with MAA. These MIPs effectively retained the imprinted doxorubicin in highly acidic (pH 1.0) solution compared to the non-imprinted polymers. When the pH increased to 6.8, which simulated intestinal fluid, the drug was released.^41^ Another successful application of such approach was exemplified by the use of pH-sensitive hydrogels from frontally polymerized *N*-isopropylacrylamide. The specific release of the imprinted gatifloxacin was observed at pH 7.4 followed by a slowdown in release upon the decrease in pH to 1.0.^42^ These effects have been confirmed by *in vivo* studies of MIPs imprinted with capecitabine based on polyhedral oligomeric silsesquioxane (POSS) and Mobil composition of matter no. 41 (MCM-41). This study expanded the existing MIP formulations suitable for peroral delivery.^43^

### Light-sensitivity

The light-controlled drug release may be another attractive option for the drug targeting. The use of near-infrared light sources, that may penetrate the tissues, in combination with up-conversive fluorophores provides a plethora of opportunities for the precise tumour therapy. Inclusion of azobenzene derivatives in the mixture of monomers is a standard routine for synthesis of photosensitive MIPs. Active groups of the compound may exist in two forms: stable *cis*-isomer and meta-stable *trans*-isomer. Upon UV-light irradiation, azobenzene isomerizes and transits from *trans*- to *cis*-form. The reverse transition may be launched by visible light or by an increase in temperature.^44^ This principle allows develop smart materials with mechanical and optical properties sensitive to light irradiation.^45,46^ An example of such a light-sensitive material is the MIP consisting of cross-linked 4-phenylazobenzoic acid (MPABA), which specifically recognizes and binds caffeine. Under UV-irradiation, the *trans*–cis isomerization leads to the release of 58.3% of bound caffeine. It may be loaded back when irradiated with 440 nm visible light.^47^ The use of multilayer supramolecular structures enables the buildup of MIPs, which would specifically release the cargo when irradiated at other wavelengths.^48^ Although the development of photosensitive imprinted nanoparticles seems feasible, the possibility of their medical use for drug delivery still has to be explored.

### Temperature-sensitivity

Another class of reactive nanomaterials is thermosensitive polymers. The release mechanism of bound molecules upon a temperature shift is similar to that for pH-sensitive polymers. Typically, they are loaded at the minimally low solution temperatures, when imprinted sites collapse and thus seal the payload inside the particle. When the temperature rises, the pores open and the imprinted molecules discharge. Such particles may be synthesized basing on the mixture of *N*-isopropylacrylamide (NIPAm), MAA and EGDMA. In this case, acrylamide derivatives take the role of the thermo-sensitive element. However, in biological systems the temperature window is narrow, which affects the efficacy of this approach. The MAA based MIPs imprinted with 4-amino pyridine effectively respond to the temperature shift. Augmentation of the temperature results in the release of the 80% of a drug compared to 60% in the control particles.^49^ In addition to small molecules, small peptides and proteins may also serve as templates for temperature-controlled release. This possibility was demonstrated for particles fabricated from cross-linked NIPAM and carrying an imprinted enzyme, lysozyme. The conformational changes were launched by the rise of temperature above 33 °C, resulting in the reversible release of the enzyme.^50^

The composition of the monomeric mixture often determines the properties of synthesized particles. Different modalities or the combination thereof may be easily obtained by simply adding the necessary compound – therapeutic, fluorescent or thermosensitive moieties. A good example of such multifunctional particles are thermosensitive fluorescent particles sized 60–200 nm and fabricated using the MAA, 2,6-bisacrylamidopyridine, and *N*-methylenbisacrylamide (NMBA) monomers imprinted with thalidomide. When the temperature raised above the critical low range temperature of the solution, the particles specifically released the *R*-enantiomer of thalidomide, which restricts the growth of cancer cells in culture. Furthermore, fluorescent labels enable easy tracing of the release spots.^51^

The versatility of MIPs is based on the wide spectrum of available monomers that can be used to achieve the required properties. A simple and effective approach has been developed for the synthesis of nanoMIPs with reversible and specific thermo- and photosensitive release of target molecules in aqueous solutions.^52^ To generate MIPs against the herbicide, 2,4-dichlorophenoxyacetic acid (2,4-D), silicon microspheres were first coated with a shell of molecularly imprinted polymers based on 4-((4-methacryloyloxy)phenylazo)pyridine and EGDMA. Next, the surface of the produced core–shell particles was additionally functionalized with “brushes” from a thermosensitive mixture of poly(*N*-isopropylacrylamide)-*b*-polyhydroxyethylmethacrylate followed by subsequent removal of the silica core. The resulting 500–600 nm size particles specifically bound 2,4-D and were stable at 37 °C temperature. However, changes in the ambient temperature, or the presence of UV-radiation, triggered the release of the target molecule. This study represented one of the first attempts to make a multifunctional carrier capable of recognizing several external stimuli.^52^

This study also confirmed the possibility of developing MIP-based carriers that specifically respond to the temperature shifts. It is tempting to speculate that one can fabricate such MIPs with the payload circulating in the bloodstream under physiological conditions but releasing the drug upon a temperature shift (disease state).

### Metal-containing MIPs for multimodal sensitivity

The polymeric nature of MIPs enables their use both as free nanoparticles and as a coating agent. The hybrid particles typically consist of a metal core and a polymer shell. The combination of functionalities of these two materials expands the versatility of the resulting MIPs and increases the precision of drug release. Such hybrid particles can also be programmed to respond to a combination of external stimuli. In this mode, a magnetic core can simultaneously act as a reporter for magnetic particle imaging (MPI) or magnetic resonance imaging (MRI), or as actuator that stimulates the release of a therapeutic agent.^53–55^

A successful use of such hybrid materials is exemplified by superparamagnetic particles coated with a thermosensitive polymer fabricated by the cross-linked NIPAM with imprinted 5-fluorouracil (5-FU) 130–150 nm size. They are able to specifically bind a therapeutic molecule and release it at temperatures above 42 °C (up to 91.17% at 45 °C), while retaining all properties of superparamagnetic core.^56^ Through rational particle design, the release temperature may be lowered to 25 °C.^57^ Alternatively, a mixture of NIPAM and cyclodextrins can be used for polymerization. In this case, more structurally complex molecules such as curcumin can be imprinted.^58^

Protocols of supramolecular chemistry enable the development of elaborate multilayer structures with complementing properties of different coatings such as a combination of MIPs and metal–organic frameworks (MOF). In this case, the polymer stabilizes MOFs in different media and prevents them from degradation.^59^ One of the first particles of this class were [Cu_3_(BTC)_2_(H_2_O)_3_]_*n*_ (HKUST-1) frameworks coated with 4-methyl phenyl dicyclohexyl ethylene (MPDE) polymer, carrying the imprinted anticancer drug, capecitabine. This modification stabilized HKUST-1 in aqueous solutions. The drug was controllably released only at moderate pH, which is convenient for oral administration. The produced 2 μm particles had a very low toxicity and a good pharmacokinetic profile, thus prolonging the circulation of capecitabine in the blood stream.^60^

The superparamagnetic structures can also be used for tracking *via* MRI or MPQ *in vivo*. Simultaneously they can generate heat when exposed to alternating magnetic field.^61,62^ Such possibility has been shown for the delivery of imprinted doxorubicin or olanzapine by core–shell particles fabricated using magnetite coated with MIPs.^63–65^ Thermal ablation may also be performed by using hybrid particles with a metal plasmon core such as gold nanorods. In this case, MIP coating performs the targeting. The example is the 40 nm by 10 nm rods coated with MIPs imprinted with sialic acid. The particles bound specifically to hepatocellular carcinoma HepG-2 cells, but not to normal hepatocytes of the L-02 cell line. Under laser irradiation at the wavelength of the surface plasmon resonance of particles (750 nm), thermal ablation of the target cells occurred. *In vitro* data was reproduced *in vivo* using a HepG-2 tumour xenograft in nude mice. After the intratumoural injection of particles, followed by irradiation with a far-red laser, the tumour volume reduced after 14 days in comparison with the unirradiated control. However, the targeting of particles to cells perhaps occurred nonspecifically due to the enhanced permeability and retention (EPR) effect.^66^ It should be noted that most of the research in this area has been carried out only within the last few years (Table 1). Thus, we can expect many more exciting discoveries in this area in the nearest future.

**Table 1:** MIP mediated drug delivery in cell and animal models

	Composition	Targeting route	Cell line	Load	Effect	
***In vitro* studies**						
1	Methacrylic acid (MAA)	EGFR	MDA-MB-468	DOX	Cytotoxic	70
	*N*-Isopropyl acrylamide (NIPAm)			Fluorescein		
	*N-tert*-Butyl acrylamide (TBAm)					
	*N*-(3-Aminopropyl)methacrylamide					
	*N*,*N*′-Methylenebisacrylamide (BIS)					
2	Zinc acrylate (ZnA)	HER-2	SK-BR-3	DOX	Cytotoxic	79
	Acrylamide (AAm)					
	Ethylene glycol dimethacrylate (EGDMA)					
3	MAA	VEGF	—	Quantum dots	Labelling	37
	NIPAm					
	TBAm					
	*N*-(3-Aminopropyl)methacrylamide					
	BIS					
4	Graphene oxide sheath	CA125	HEK293	DOX	Cytotoxic	28
	Dopamine (DA)		SMMC-7721			
	Aminopropyltriethoxysilane (APS)					
5	MAA	Folate receptor	MDA-MB-231	Paclitaxel	Cytotoxic	80
	EGDMA					
6	SiO_2_ core	Sialic acid	HepG-2	Fluorescein	Labelling	81
	Tetraethyl orthosilicate (TEOS)		MCF-7			
7	MAA	Light irradiation	HeLa	Carbazole	Cytotoxic	46
	EGDMA		MCF-7			
	2,2′-Azoisobutyronitrile (AIBN)					
8	AAm	Light irradiation	HeLa	Sunitinib	Cytotoxic	82
	Glycidyl methacrylate (GMA)		MCF-7			
	AIBN		ARO			
	EGDMA		WRO			
9	Magnetic core	Magnetic field	PC-3	DOX	Cytotoxic	54
	Oligo-(ethylene glycol) methyl					
	Bis(ethylene glycol) methylacrylate (MEO_2_MA)					
	MAA					
	Acrylamide					
	EGDMA					
10	Magnetic core	Magnetic field	PC-3	DOX	Cytotoxic	55
	AAm					
	EGDMA					
11	MMA	*S. aureus*		DOX	Antibacterial	29
	Cetyl alcohol (CA)					
	AIBN			QDZ		
	EGDMA					
12	AAm	Lpp20	*H. pylori*	Amoxicillin	Antibacterial	30
	BIS					
13	AAm	Polysaccharide capsule	*P. aeruginosa*	DOX	Antibacterial	31
	BIS			Fluorescein		

***In vivo* studies**						
1	MAA	—	—	*S*-amlodipine	Calcium channel blocker	7
	4-Methyl phenyl dicyclohexyl ethylene (MPDE)					
	EGDMA					
2	AAm	—	—	Capecitabine	Cytotoxic	43
	2-Acrylamido-2-methylpropanesulfonic acid (AMPS)					
	Polyhedral oligomeric silsesquioxanes (POSS)					
	Mobil composition of matter no. 41 (MCM-41)					
	EGDMA					
3	Si core	CD59	MCF-7	DOX	Cytotoxic	83
	TFMA			CE6		
	NIPAm		LoVo			
	TIBAm					
	BIS			Quantum dots (QDs)		
4	ZIF-8 MOF core	CD59	MCF-7	DOX	Cytotoxic	84
	Dimethylaminoethyl methacrylate (DMAEMA)					
	NIPAm					
	TBAm					
	Trifluoromethyl acrylate (TFMA)					
	*N*,*N*′-Diacrylylcystamine (BAC)			QDs		
5	NIPAm	p32	4T1	DOX	Cytotoxic	85
	TBAm					
	Trifluoromethyl acrylate (TFMA)					
	BIS					
6	AAm	p32	4T1	FAM	Cytotoxic	26
	BIS		BxPC-3 c	Methylene blue		
7	MAA	β2 microglobulin (B2M)	EJp16	Dasatinib	Cytotoxic	72
	*N*-Isopropyl acrylamide (NIPAm)					
	*N-Tert*-butyl acrylamide (TBAm)			DyLight 800		
	*N*-(3-Aminopropyl)methacrylamide					
	BIS					
8	ZnA	Fn14	BxPC-3	Bleomycin	Cytotoxic	86
	Vinylbenzeneboronic acid (VPBA)					
	EGDMA					
9	*N*-Acryloyl-l-phenylalanine (APA)	Folate receptor	HeLa	Vinblastine	Cytotoxic	87
	*N*-Acryloyl-l-lysine (ALys)					
	*N*,*N*′-bis(Acryloyl)cystamine (BACy)					
10	Gold nanorods core	Sialic acid	HepG-2	Fluorescein	Thermoablation	66
	TEOS					
11	AAm	EGFR	HeLa	QDs	Labeling	27
	BIS					
12	SiO_2_ core	HER-2	SkBr3	—	Inhibition of HER2 activation	73
	TEOS					
13	HEMA	Contact lenses	—	Ciprofloxacin	Antibacterial	24
	Methacryloxy propyl tris (trimethylsiloxy) silane (TRIS)					
	Polyvinylpyrrolidone (PVP)					
14	2-Hydroxyethylmethacrylate (HEMA)	Contact lenses	—	Ketotifen	Antihistamine	88
	Acrylic acid (AA)					
	Acrylamide (AAm)					
	AIBN					
	*N*-Vinyl 2-pyrrolidone (NVP)					
	Polyethylene glycol (200) dimethacrylate (PEG200DMA)					
15	*N*,*N*-Diethylacrylamide (DEAA)	Contact lenses	N/A	Timolol	Beta blocker	23
	MAA					
	EGDMA					

## The next generation MIPs – from solution to solid phase

**Fig. 3:**
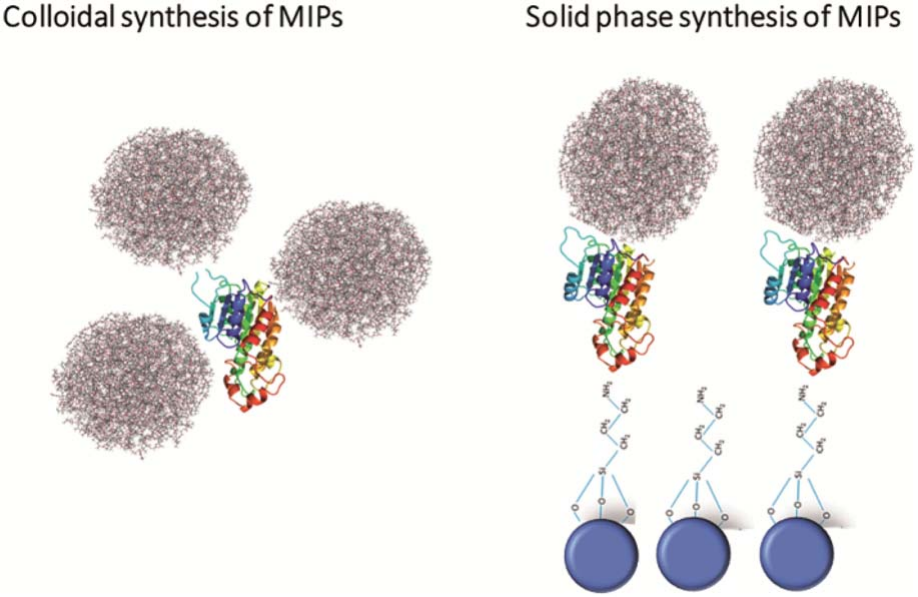
Strategies for MIP synthesis. Solid phase synthesis enabled the imprinting of a much broader spectrum of molecules when compared to standard protocols in solvents. Shown are the protein templates (colour ribbons) covalently bound on the surface of silanized glass beads (blue circles) or slides. The polymerization reaction occurs upon addition of the monomer solution together with the initiator, and operates as the reaction centres. Finally, specific MIP nanoparticles carrying the template imprint (fluffy balls) are eluted from the solid phase by increasing the temperature the solvent. This approach provides uniformity and a high specificity of MIPs compared to the colloidal synthesis, which yields a mixture of MIPs with different affinity.

Although the transition to the non-covalent binding between polymer and template has enabled the use of MIPs for the recognition of cell surface markers, it had one significant drawback – lack of generic, versatile, scalable and cost-effective approach for their manufacturing. This issue has been resolved by the introduction of a solid-phase MIPs synthesis, developed by the Piletsky group in 2013.^1^ It was the result of rethinking the concept of polymer preparation in line with automated protocols used in synthesis of peptides and oligonucleotides. The target molecules in this case were covalently immobilized on the solid phase, *e.g.*, glass beads. Next, monomers were polymerized around the template, thereby performing imprinting. After the removal of the unreacted monomers and nonspecific polymer particles, high affinity polymer particles were subject to a temperature-based affinity separation, being simply eluted from the solid phase template by means of a temperature increase of the solvent. The resulting 100–200 nm size MIPs carried the imprints of target molecules and were able to specifically recognize them in different settings. The usability of this strategy was first demonstrated for melamine, vancomycin and various peptides as targets.^1,67^ Furthermore, this method was modified for the imprinting of DNA, proteins, and viruses.^68^ The obtained particles were apparently non-toxic, did not change the cellular metabolism, and were efficiently internalized by cells *via* the endocytosis route. The latter was shown *in vitro* on various cancer cell lines including HaCaT, MEFs, and HT1080.^69^ The key advantages of this method compared to the emulsion-based one include a more homogeneous distribution of binding site affinities (deriving from the orientation of the template on the solid phase), better accessibility of binding sites, and the increased affinity for target molecules even in a complex molecular environment. Additionally, this approach enables the recognition of an extremely wide range of target molecules and the possibility of a complete automation of the synthetic process. The implementation of this synthesis method resulted in a dramatically increased speed of fabrication of MIPs against various cell surface markers. Thus, they became a powerful targeting nanoagents (Fig. 3).

### NanoMIPs as carriers for the targeted delivery of drugs *in vivo*

One of the most prospective therapeutic applications of MIPs resides in the possibility of systemic treatment of various malignancies. MIPs possess all the necessary properties to become the next major platform for the development of cancer therapy due to their versatile nature and ability to mediate specific drug delivery. In addition to the ability of specific recognition of various cell surface markers in complex environments, MIPs are biocompatible and can be programmed to release their therapeutic cargo in response to specific external stimuli.^18^ The first use of MIPs for *in vivo* cancer targeting was demonstrated by the Cuschieri's group. An oligopeptide corresponding to a fragment of the VEGF protein was used as a template for MIPs fabrication. The obtained MIPs were additionally modified by fluorescent quantum dots that were covalently immobilized on their surface. The obtained 171 nm size nanoparticles had a statistically significant tendency to accumulate near VEGF-positive cells in the WM-266 melanoma cell line xenografted into zebrafish embryos. These results confirmed the theoretical possibility of using nanoMIPs for tumour therapy. However, the efficiency of the payload delivery was not demonstrated.^37^ In the same year, the use of MIPs as therapeutic agents was shown in a collaborative study by the Barlev and Piletsky groups. Using breast cancer human cell lines MDA-MB-468 and SKBR3 as *in vitro* models, the authors have shown that the EGFR-imprinted fluorescein-labelled nanoMIPs with size 150–200 nm specifically recognized MDA-MB-468 EGFR-positive cells, but not SKBR3 EGFR-negative cells. Due to an additional imprinting with doxorubicin in the core of EGFR-specific nano-MIPs, they reduced the survival rate of MDA-MB-468 EGFR-positive cells by arresting their cell cycle and increasing apoptosis. Importantly, no appreciable effect of doxorubicin-loaded EGFR-nanoMIPs was detected in SKBR3 EGFR-negative cells.^70^ The *in vivo* use of nano-MIPs obtained by solid-phase synthesis has been recently demonstrated by Macip's group with the aim to target senescent cells in immune-deficient mice. By using double-imprinted 133 nm size nano-MIPs against a β2 microglobulin (B2M) epitope, one of the cell surface markers of premature senescence,^71^ selective nano-MIPs were successfully synthesized in the presence of the senolytic tyrosine-kinase inhibitor, dasatinib. Fluorescent tagging showed selective binding of B2M-nanoMIPs to senescent cells, proportional to the amount of B2M protein expressed on their surface.^72^ Importantly, the treatment of B2M-positive EJ bladder cancer cells with these MIPs (B2M-MIPs) significantly reduced the proliferation of cancer cells.

The demonstrated synthesis of standardized nano-MIPs capable of specific delivery of therapeutic or reporter molecules has stimulated interest for their *in vivo* application. In addition to these studies, the work of Liu's group also proved the applicability of MIPs as potential therapeutics. The study demonstrated the effective binding of hybrid silica-based nanoparticles coated with *N*-glycan MIPs to HER2-positive SKBR3 breast cancer cells. Treatment of cells with MIPs blocked the receptor dimerization and hence prevented subsequent triggering of the corresponding signaling pathways. The latter significantly reduced the proliferation rate of HER2-positive SKBR3 cells *in vitro* and in mouse xenografts *in vivo*.^73^

## Conclusions

**Fig. 4:**
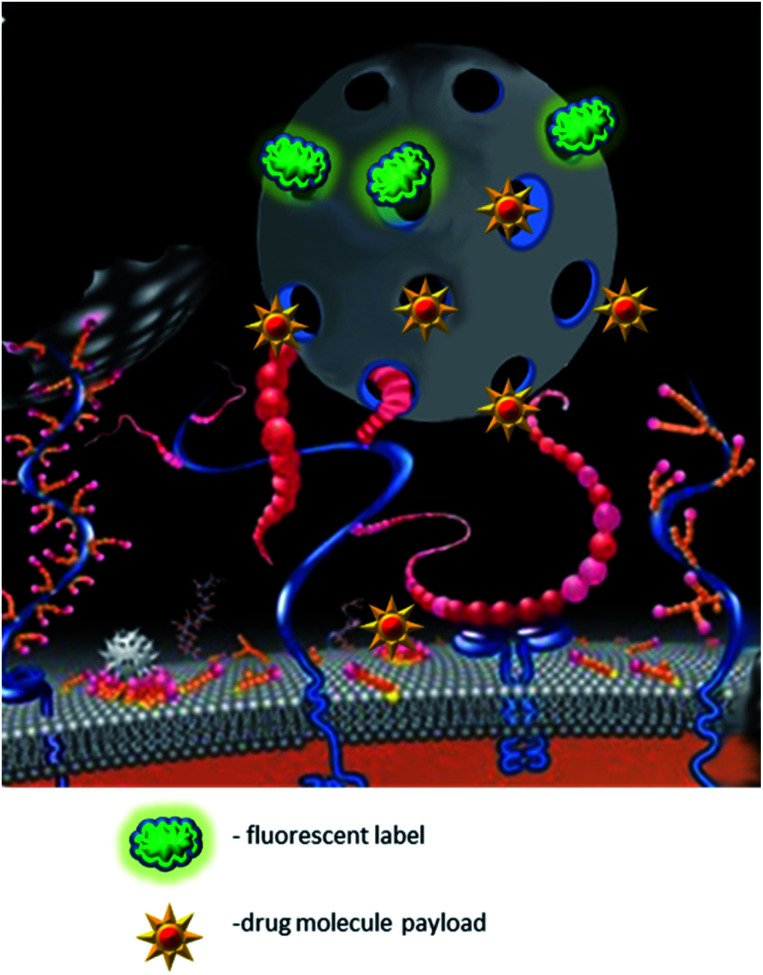
Payload specific delivery by MIPs. The payload delivery to specific cells can be mediated by nanoMIPs (depicted as a grey ball). Multiple imprints of nanoMIPs can extend their functionality. For example, nanoMIPs imprinted against the cell surface receptors (shown as red and blue strings) can also carry the pharmaceutical payload (*e.g.*, doxorubicin, shown as stars) and at the same time be labelled with a fluorescent dye (*e.g.*, Cy5 depicted as green clouds) for subsequent visualisation.

In summary, nano-MIPs clearly represent a new promising nanomaterial-based approach for targeted delivery of different payloads. By varying the monomer composition, it is possible to fabricate biocompatible, potentially even biodegradable, or stimulus-sensitive nanoparticles. The specificity of the drug delivery may be enhanced by using template proteins that are unique for target cells or overexpressed on their surface, *e.g.*, EGFR or PDGFR in specific epithelial cancers. Introduction of synthetic linear peptides as templates that reflect the structure of target epitopes has simplified the synthesis process, as well as enhanced the reproducibility of the physical properties of the particles being developed (Fig. 4). Fabrication of nano-MIPs by the double imprinting method provides a new technological breakthrough allowing different combinations of drugs while keeping the precision of specific delivery.

There are several features that largely determine the success of a potential drug delivery platform in clinic such as biocompatibility, specificity and compatibility with its potential cargo. The vehicle should demonstrate high safety profile and ideally must be completely cleared off the organism shortly after performing its function. On the contrary, when systemically injected nanoparticle-carriers should evade the reticuloendothelial system to effectively infiltrate the target tissues. The non-selective activity of most drugs when introduced systemically, limits their efficacy due to various adverse effects. This problem may be solved either by exogenous control of the precise release of therapeutic cargo from the vehicle (*e.g.* application of magnetic fields), or by guiding the nanocarrier to the tissue of interest by immobilizing specific recognition/targeting molecules on its surface. Since most disorders are caused by breakdown in more than one regulatory mechanism, the ideal platform should be able to deliver several therapeutic molecules of different size and nature.

Having said that, the unique biophysical properties of MIPs match all the criteria mentioned above and make them a highly perspective platform for clinical applications. The nanoMIPs carriers exhibit a good biocompatibility profile. The possibility for multiple imprints on their surface makes them a versatile tool for specific and simultaneous delivery of several therapeutics and molecular probes. Furthermore, nanoMIPs can be employed as a platform for the development of smart materials. For instance, the use of light-sensitive or photo-responsive monomers allows for the activation of the drug release *via* external stimuli. Addition of sensor molecules to the MIP composition would likely help visualize the site of the drug release, or even help measure the kinetics of this process.^41^ Importantly, polymer-based materials not only provide a means for the controlled and gradual release of a drug at a particular site, but also augment the specificity of targeting. The surface of nanoparticles can be easily functionalized with the targeting molecules such as antibodies, aptamers, or trap-receptors, that would guide them to the malignant tissue. The insertion of a metal core into MIPs may provide additional features which could be used for *in vivo* therapeutic applications. *E.g.*, magnetic nanoparticles may be used for local hyperthermia in the body when positioned in an alternating magnetic field. Furthermore, silver and gold plasmonic particles may generate heat when irradiated by light beams of specific wavelengths.^74^ The development of polymeric nanoparticles, which would synergistically combine the advantages of both materials, will likely build a new technological platform for diagnostics and therapy.^14^ Evidently, the success of numerous studies made over the past two decades have gradually shifted the paradigm of MIPs employability from sheer cargo transporters to smart carriers capable of recognition and specific delivery of therapeutics to the target cells (Table 1).

Currently, the key limitation for the broad application of nanoMIPs in biomedicine instead of conventional antibodies is the necessity of careful selection of the target peptide for imprinting, which should represent a biologically sound epitope *in vivo*. In this respect, a recent study from the Piletsky group has described an approach how to do an unbiased mapping of the biologically relevant protein epitopes using MIPs.^75^ Another obstacle to the wider use of nanoMIPs in diagnostics is the sensitivity of imprints on the surface of nanoMIPs to any chemical modification after their removal from the solid surface. It significantly narrows the applicability of the nanoMIP-based delivery platforms by limiting the functionalization of the ready-made particles by fluorescent labels or additional polymer coating. Finally, there is very little information on the behaviour of nanoMIPs *in vivo*, such as their pharmacokinetics, bioavailability, toxicity *etc.* Despite the obvious importance of such information for the translation aspect of MIPs applicability, this gap started to be patched only recently. *In vivo* studies on the bioavailability and compatibility of MIPs immediately highlighted several major limitations of the use of synthetic polymers in systemic delivery. One of such limitations is the formation of a protein corona that sterically blocks the active binding sites of target molecules and activates the complement.^76–78^ However, it is important to note that this problem is intrinsic to nanoparticle-based systems in general. Successful solving this problem is a matter of future research.

## Conflicts of interest

S. A. P. is the founder and F. C. is the head of chemistry in MIP Diagnostics Ltd, UK.

## Supplementary Material
